# Extracellular CIRP induces acute kidney injury via endothelial TREM-1

**DOI:** 10.3389/fphys.2022.954815

**Published:** 2022-09-29

**Authors:** Sara Siskind, Fangming Zhang, Max Brenner, Ping Wang

**Affiliations:** ^1^ Department of Surgery, Zucker School of Medicine at Hofstra/Northwell, Manhasset, NY, United States; ^2^ Center for Immunology and Inflammation, The Feinstein Institutes for Medical Research, Manhasset, NY, United States; ^3^ Elmezzi Graduate School of Medicine, Manhasset, NY, United States

**Keywords:** acute kidney injury, inflammation, TREM-1, eCIRP, DAMP, endothelial cell

## Abstract

**Introduction:** Acute kidney injury is associated with elevated serum levels of extracellular cold-inducible RNA-binding protein (eCIRP), a damage-associated molecular pattern released during ischemia/reperfusion injury, hemorrhagic shock, and sepsis. It is unknown if circulating eCIRP and eCIRP-induced activation of receptor triggering receptor expressed on myeloid cells-1 (TREM-1), expressed on endothelial cells, play an important role in the pathogenesis of AKI.

**Methods:** Male B6 wild-type (WT) and TREM-1^−/−^ mice were subjected to intravenous injection of recombinant murine (rm) CIRP. Serum, urine, and renal tissue were collected 6 h later for analysis. Additionally, primary human renal glomerular endothelial cells (HRGEC) were stimulated *in vitro* with rmCIRP after pretreatment with M3, a novel inhibitory peptide of TREM-1, or vehicle. Supernatants and cells were collected 20 h after stimulation.

**Results:** After injection with rmCIRP, WT mice had a significant increase in serum levels of BUN, creatinine, and NGAL compared to control. Additionally, NGAL was significantly increased in the urine of rmCIRP-injected mice, suggesting that circulating eCIRP can directly induce AKI. The levels of TREM-1 mRNA in the kidneys, as well as soluble (s) TREM-1 released into the serum and urine, were significantly increased in rmCIRP-injected mice. TREM-1^−/−^ mice injected with rmCIRP had attenuated AKI, indicated by significantly decreased serum BUN, creatinine, and NGAL, and renal mRNA expression of NGAL and KIM-1 compared to WT mice. TREM-1^−/−^ mice also had attenuated endothelial activation, with decreased mRNA and protein expression of ICAM-1 in renal tissue. HRGEC stimulated with rmCIRP *in vitro* had significant increases in cytokine production and sTREM-1 release, which was attenuated in cells treated with M3.

**Conclusion:** Activation of renal TREM-1 with circulating eCIRP is sufficient to cause AKI. Elevated levels of eCIRP may be critical for the development of AKI under conditions such as ischemia/reperfusion injury, hemorrhagic shock, and sepsis. Mice deficient in the TREM-1 receptor have attenuated AKI and reduced endothelial cell activation after injection of rmCIRP. TREM-1 inhibition with M3 attenuates HRGEC activation after eCIRP stimulation. Targeting eCIRP activation of TREM-1 may provide a novel and effective treatment for AKI.

## Introduction

Acute kidney injury (AKI) is a frequent complication among hospitalized patients, defined as a sudden decrease in kidney function over a period of hours to days (Coca et al., 2009; Rewa and Bagshaw, 2014; Makris and Spanou, 2016). This injury encompasses a wide range of physiologic impairments, from small changes in serum biochemical markers and decreased urine output to kidney failure requiring dialysis, and is associated with substantial short and long-term morbidity and mortality (Coca et al., 2009; Rewa and Bagshaw, 2014; Vijayan, 2021). AKI can result from a variety of insults to the kidney, including hypovolemia secondary to hemorrhage and sepsis, or renal ischemia/reperfusion (RIR) injury (Basile et al., 2012; Makris and Spanou, 2016). These etiologies share a common pathophysiology in that when blood flow to the kidney is decreased or absent, there is an insufficient oxygen and nutrients delivered to the tubular epithelial cells, causing damage and subsequent apoptosis or necrosis. These damaged cells release damage-associated molecular patterns (DAMPs) which activate the innate immune response and amplify cellular injury through the release of pro-inflammatory cytokines (Thurman, 2007; Bonventre and Yang, 2011; Basile et al., 2012; Peerapornratana et al., 2019). Regardless of the etiology, care for AKI remains supportive, with fluid administration and/or diuretics, avoidance of nephrotoxic agents, correcting the underlying etiology, and renal replacement therapy when necessary (Kaushal and Shah, 2014; Godin et al., 2015; Peerapornratana et al., 2019). There is currently no effective treatment aimed at reducing renal injury after an insulting event (Jo et al., 2007).

Cold inducible RNA binding protein (CIRP) is a cold shock protein that under normal conditions is located intracellularly and regulates protein expression (Qiang et al., 2013). During sepsis and hemorrhagic shock, CIRP is released into the extracellular space, acting as a DAMP and aggravating the inflammatory response (Qiang et al., 2013; Aziz et al., 2019). Extracellular CIRP (eCIRP) release has also been implicated in the progression of AKI following RIR injury through activation of triggering receptor expressed on myeloid cells-1 (TREM-1), a pattern recognition receptor (PRR) involved in the innate immune response (Gibot et al., 2004; Cen et al., 2016; Siskind et al., 2022). Both mice genetically deficient in TREM-1 and treated with M3, a novel peptide designed to inhibit the interaction of eCIRP with TREM-1, had attenuated renal injury after RIR (Denning et al., 2020a; Siskind et al., 2022). Recently, we found that eCIRP can independently induce a systemic inflammatory response akin to sepsis through activation of TREM-1, however, the resultant effects on kidney function have not been thoroughly explored (Denning et al., 2020a). Further characterization of the direct effects of eCIRP on the development of AKI *via* TREM-1 will improve our understanding of the pathophysiology of this disease and target this novel pathway for therapeutic development.

Both eCIRP and TREM-1 are upregulated in AKI, and blockade of TREM-1 attenuates AKI severity (Campanholle et al., 2013; Lo et al., 2014; Cen et al., 2016; Tammaro et al., 2016; Tammaro et al., 2017; Siskind et al., 2022), giving rise to the hypothesis that eCIRP directly induces renal inflammation *via* activation of TREM-1. In this study, we have demonstrated that circulating eCIRP can independently induce AKI through interaction with TREM-1. We further propose a mechanism of action by providing evidence that eCIRP activates TREM-1 expressed on renal endothelial cells, inducing endothelial cell activation and release of inflammatory and chemoattractant factors. Inhibition of renal glomerular endothelial cells with M3 protects cells from eCIRP-induced activation.

## Materials and methods

### Experimental animals

C57BL/6 male mice were purchased from Charles River Laboratories. The TREM-1^−/−^ mice were generated by the trans-NIH Knockout Mouse Project (KOMP) and obtained from the KOMP Repository, University of California, Davis, lines maintained by in-house breeding. All experiments used age-matched mice at 8–12 weeks old. Only male mice were used in this study based on evidence that there are sex-specific differences in the immune response to sepsis (Angele et al., 2014). It has been reported that female sex hormones can be protective against the overwhelming inflammatory response in sepsis, and therefore males produce increased levels of inflammatory cytokines (Zellweger et al., 1997; Angele et al., 2014; Kuo, 2016). All experiments involving live animals were evaluated and approved by the Institutional Animal Care and Use Committee (IACUC) at the Feinstein Institutes for Medical Research.

### *In vivo* administration of recombinant murine (rmCIRP)

WT and TREM-1^−/−^ mice were anesthetized with inhaled isoflurane on a surgical plane and restrained in prone position. rmCIRP (produced in our lab as previously described) at a dose of 3 mg/kg BW (100 μL) was administered *via* retro-orbital injection using a 26G × 1/2″ needle on a 1 ml syringe (Qiang et al., 2013; Denning et al., 2020a). At 6 h after rmCIRP injection, mice were anesthetized, and blood, renal tissue, and urine were collected for analysis. The kidneys were frozen in liquid nitrogen and stored at −80°C for quantitative PCR and protein analysis.

### Cell culture, stimulation, and treatment

Primary Human glomerular renal endothelial cells (HRGECs) were purchased from ScienCell Research Laboratories. The cells were maintained in complete endothelial cell medium (ScienCell) in fibronectin coated plates (Invitrogen, Thermo Fischer Scientific) at 37 °C in a 5% CO_2_ humidified incubator, per culturing instructions. All experiments were performed between passages 2 and 5. Cells were stimulated with 1 μg/ml of rmCIRP 30 min after pretreatment with 10 μg/ml of M3 (RGFFRGG; GenScript) or PBS vehicle. Supernatants from control and stimulated cells were collected at 20 h after stimulation, and cells were lysed for intracellular protein or mRNA analysis.

### Determination of BUN and creatinine

Serum levels of BUN and creatinine were determined using specific colorimetric enzymatic assays according to manufacturer instructions (Pointe Scientific).

### ELISA and cytokine array

Supernatant, serum, or urine protein was analyzed by ELISA kits specific for IL-6 (BD Biosciences), ICAM-1 (R&D Systems), NGAL, and TREM-1 (Invitrogen, Thermo Fischer Scientific) according to manufacturers’ instructions. HRGEC were dissolved in 100 μL of in-house prepared RIPA lysis buffer. The kidney tissue was crushed in liquid nitrogen and ∼100 mg of powdered tissue was dissolved in 500 μl of in-house prepared RIPA lysis buffer. All samples were sonicated on ice and protein concentration was determined by Bradford protein assay reagent (Bio-Rad). 40 μg of protein from HRGEC was used for assessment of IL-6 and 50 μg of kidney protein was used for assessment of ICAM-1 using respective ELISA kits. Supernatant from HRGEC was analyzed for 105 cytokines using a proteome profiler cytokine array (R&D Systems) per manufacturer’s instructions and resultant pixel density was calculated using ImageJ software.

### Isolation of the mRNA and real-time quantitative reverse transcription PCR

Approximately 100 mg of kidney tissue powder were dissolved in lysis buffer from Illustra RNAspin Mini RNA Isolation kit (GE Healthcare) and sonicated on ice. RNA was extracted using the same kit according to manufacturer instructions and subsequently reversed-transcribed into cDNA using MLV reverse transcriptase (Applied Biosystems, Thermo Fisher Scientific). The PCR reaction was performed in a final volume of 24 μL containing 4 μg cDNA, 0.08 μmol of forward and reverse primers, 10 μL SYBR Green PCR Master Mix (Applied Biosystems, Thermo Fisher Scientific), and 11 μL nuclease-free water. cDNA was amplified using a Step One Plus real-time PCR machine (Applied Biosystems, Thermo Fisher Scientific). Mouse β-actin was used for normalization and relative expression of mRNA was calculated using the ΔΔCT method. Results were reported as fold change in comparison with the control mice. Primer sequences are: β-actin: Forward: CGT​GAA​AAG​ATG​ACC​CAG​ATC​A, Reverse: TGG​TAC​GAC​CAG​AGG​CAT​ACA​G, TREM-1: Forward: CTA​CAA​CCC​GAT​CCC​TAC​CC, Reverse: AAA​CCA​GGC​TCT​TGC​TGA​GA, NGAL: Forward: CTC​AGA​ACT​TGA​TCC​CTG​CC, Reverse: TCC​TTG​AGG​CCC​AGA​GAC​TT, KIM-1: Forward: TGC​TGC​TAC​TGC​TCC​TTG​TG, Reverse: GGG​CCA​CTG​GTA​CTC​ATT​CT, ICAM-1: Forward: GGG​CTG​GCA​TTG​TTC​TCT​AA, Reverse: CTT​CAG​AGG​CAG​GAA​ACA​GG.

### Statistical analysis

Data represented in the figures are expressed as mean ± SEM. All data have been tested for normality using the Kolmogorov-Smirnov test of normality. Normally distributed data were analyzed using 1-way ANOVA for comparison among multiple groups, and the significance of differences between individual groups was determined by Tukey’s method. Student’s t-test was utilized for 2-group comparisons. The specific tests used for each graph are identified in the figure legends. All statistical analyses were conducted using Prism (GraphPad by Dotmatics), and the statistical significance threshold was set at *p* <0.05 between study groups.

## Results

### Circulating extracellular CIRP induces acute kidney injury and upregulates renal triggering receptor expressed on myeloid cells-1

**FIGURE 1:**
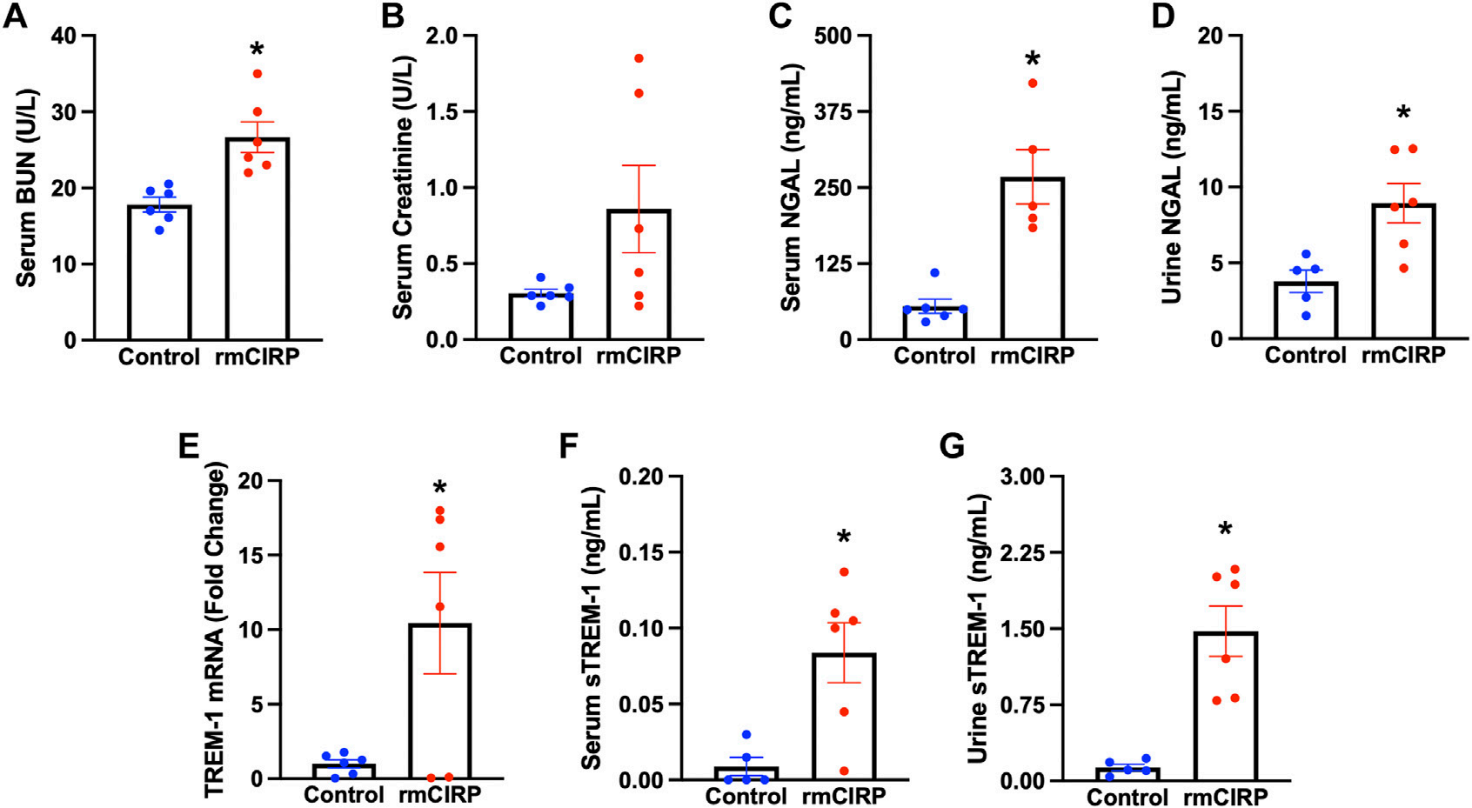
eCIRP induces AKI and TREM-1 activation. Adult C57BL/6 mice were randomly assigned to normal control or intravenous injection with recombinant murine (rm) CIRP. At 6 h after the injection, serum levels of **(A)** BUN and **(B)** creatinine (*p* = 0.08) were determined using specific colorimetric enzymatic assays, *n* = 6 mice per group. **(C)** Serum and **(D)** urine levels of NGAL were measured by ELISA, *n* = 5–6 mice per group. **(E)** Renal mRNA expression of TREM-1, and serum **(F)** and **(G)** urine levels of sTREM-1 were measured by RT-PCR and ELISA, respectively, *n* = 6 mice per group. All groups were compared using Student’s t-test. *p < 0.05 vs. control. Data are expressed as mean ± SEM.

To determine whether eCIRP is sufficient to induce AKI, we subjected WT mice to i. v. injection of rmCIRP and collected serum, urine, and kidney tissue 6 h later. Serum BUN was increased by 52% and creatinine was increased by 180% in mice injected with rmCIRP compared to control (Figures 1A,B). Additionally, NGAL protein expression, a biomarker for AKI (Soni et al., 2010), was increased by 389% in the serum and 136% in the urine compared to control (Figures 1C,D). We then evaluated if the eCIRP-induced AKI was associated with an increase in TREM-1 expression. After injection with rmCIRP, mice had a 10-fold increase in renal TREM-1 mRNA expression comparted to control (Figure 1E). Next, soluble TREM-1, the extracellular domain of TREM-1 that is cleaved and released into circulation during sepsis and sepsis-associated AKI and serves a marker of TREM-1 activation (Su et al., 2011; Jolly et al., 2021), was evaluated in the serum and urine. Mice with eCIRP-induced AKI had a 9.3-fold increase in serum sTREM-1 and an 11.3-fold increase in sTREM-1 excreted in urine (Figures 1F,G). These data indicate that eCIRP is sufficient to induce AKI, and that eCIRP-induced AKI is associated with upregulation and activation of TREM-1.

### Triggering receptor expressed on myeloid cells-1 deficiency attenuates extracellular CIRP-induced acute kidney injury

**FIGURE 2:**
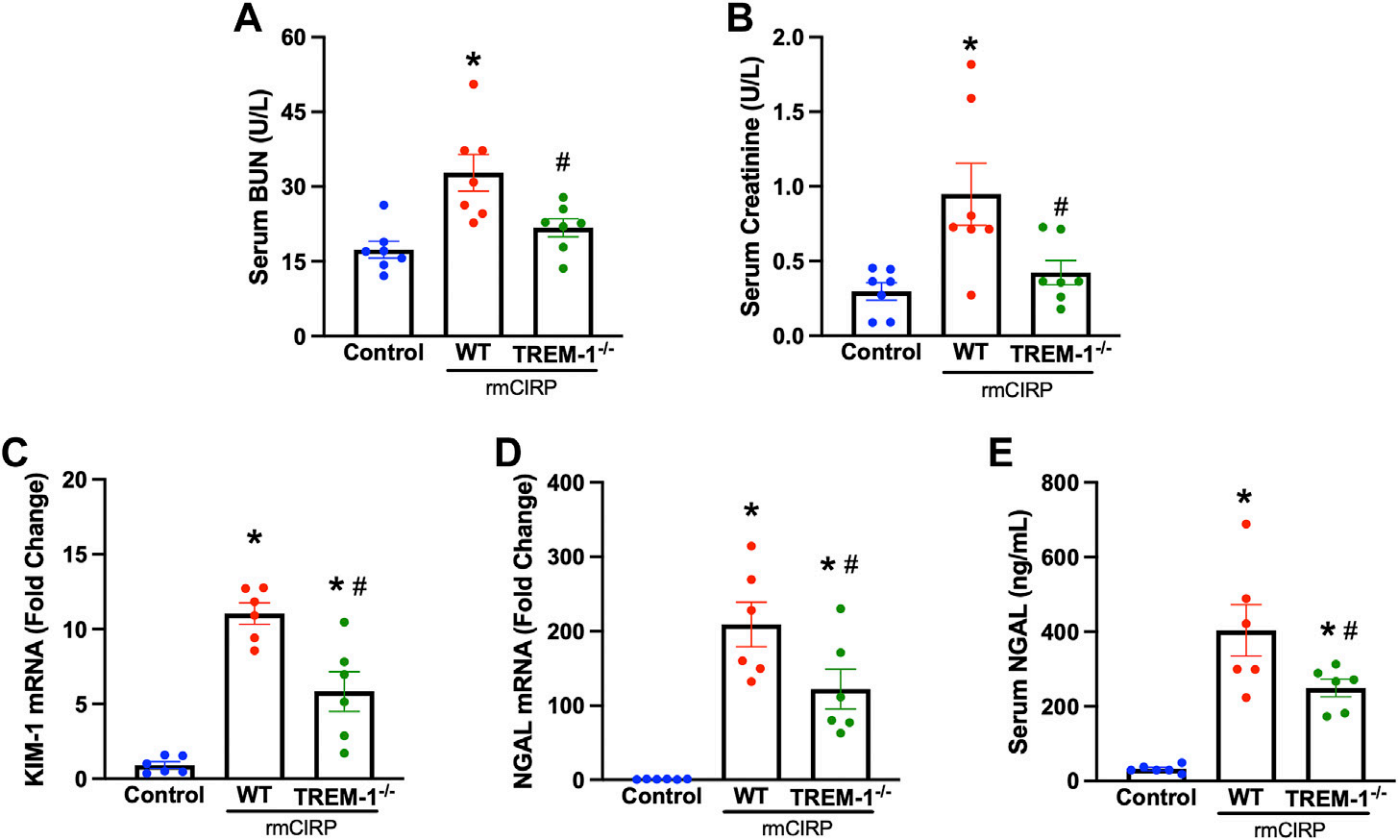
TREM-1 mediates eCIRP-induced AKI. Adult C57BL/6 and TREM-1^−/−^ mice were randomly assigned to control or rmCIRP injection. At 6 h after injection, serum levels of **(A)** BUN and **(B)** creatinine were determined using specific colorimetric enzymatic assays. Renal mRNA expression of **(C)** KIM-1 and **(D)** NGAL were measured by RT-PCR, and **(E)** serum NGAL was determined using ELISA, *n* = 6-7 mice per group. All groups were compared by one-way ANOVA and Tukey’s method. *p < 0.05 vs. control, ^#^ *p* < 0.05 vs. WT. Data are expressed as mean ± SEM.

To further verify the role of TREM-1 in AKI, we evaluated if mice deficient in TREM-1 had attenuated AKI after injection with CIRP. TREM-1^−/−^ mice and WT mice received the same dose of IV rmCIRP and collection took place 6 h later. Compared to WT mice that received CIRP, TREM-1^−/−^ mice had a 33.6% reduction in serum BUN and a 55.4% reduction in serum creatinine (Figures 2A,B). Renal mRNA expression of KIM-1, another marker of AKI (Dase et al., 2022), was evaluated in the kidneys of TREM-1^−/−^ mice and was noted to have a 47.1% decrease compared to WT mice (Figure 2C). Additionally, after rmCIRP injection, TREM-1^−/−^ mice had a 41.6% reduction in renal tissue NGAL mRNA expression and a 38.3% decrease in serum NGAL compared to WT mice (Figures 2D,E). eCIRP administration did not result in measurable histological abnormalities (data not shown). These data demonstrate that mice deficient in TREM-1 are protected from eCIRP-induced AKI, and suggest that the eCIRP/TREM-1 pathway likely plays a role in the development of AKI after a proinflammatory insult.

### Extracellular CIRP activates renal endothelial cells *via* triggering receptor expressed on myeloid cells-1

**FIGURE 3:**
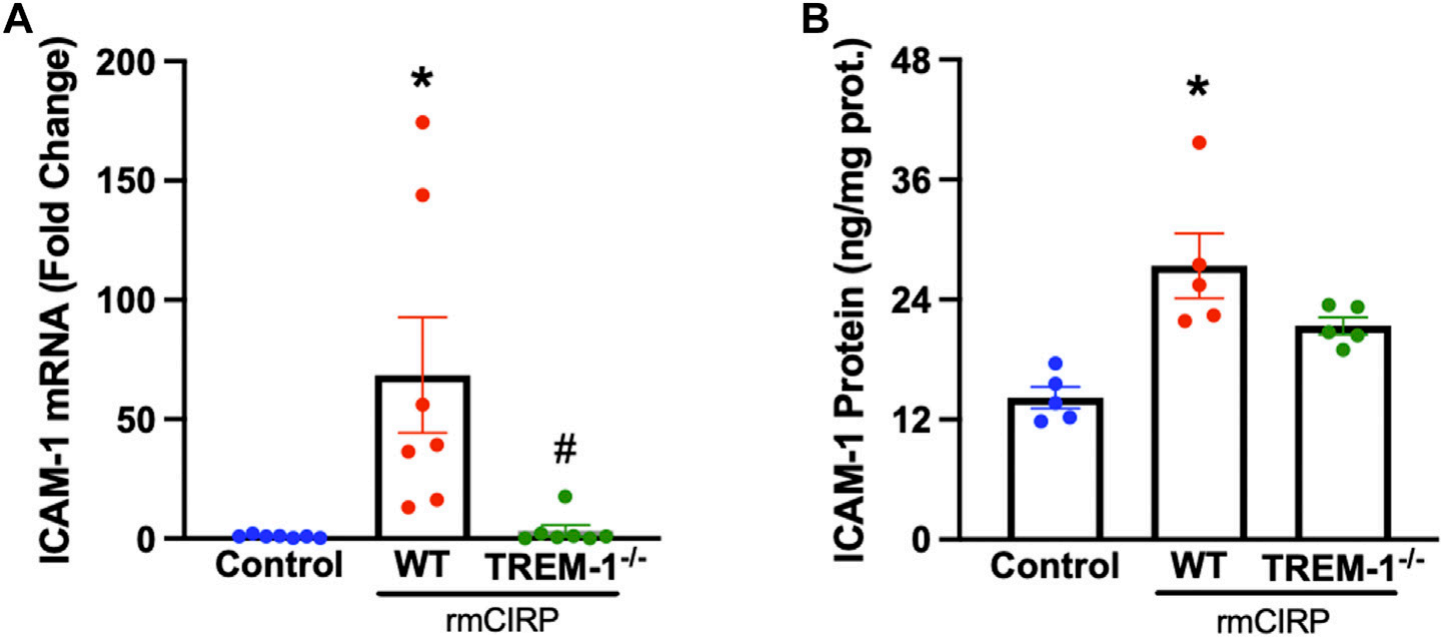
rmCIRP activates renal endothelial cells via TREM-1. Adult C57BL/6 and TREM-1^−/−^ mice were randomly assigned to control or rmCIRP injection. At 6 h after injection, renal **(A)** mRNA and **(B)** protein levels of ICAM-1 were measured using RT-PCR and ELISA, respectively, *n* = 5–7 mice per group. All groups were compared by one-way ANOVA and Tukey’s method. *p < 0.05 vs. control, ^#^ *p* < 0.05 vs. WT. Data are expressed as mean ± SEM.

TREM-1 activation on endothelial cells was recently discovered to play a role in the progression of sepsis (Jolly et al., 2018). To determine if endothelial activation within the kidneys during AKI is mediated through the interaction of TREM-1, TREM-1^−/−^ mice and WT mice were injected with rmCIRP to induce AKI and the renal tissue was evaluated for ICAM-1 expression. The renal tissue of WT mice with CIRP-induced AKI had a 100-fold increase in ICAM-1 mRNA expression and a 2-fold increase in ICAM-1 protein compared to that of control. In TREM-1^−/−^ mice, however, ICAM-1 mRNA and protein expression were reduced by 98% and 22%, respectively, compared to WT mice (Figures 3A,B).

### The novel triggering receptor expressed on myeloid cells-1 inhibitor M3 attenuates CIRP-induced renal endothelial cell activation

**FIGURE 4:**
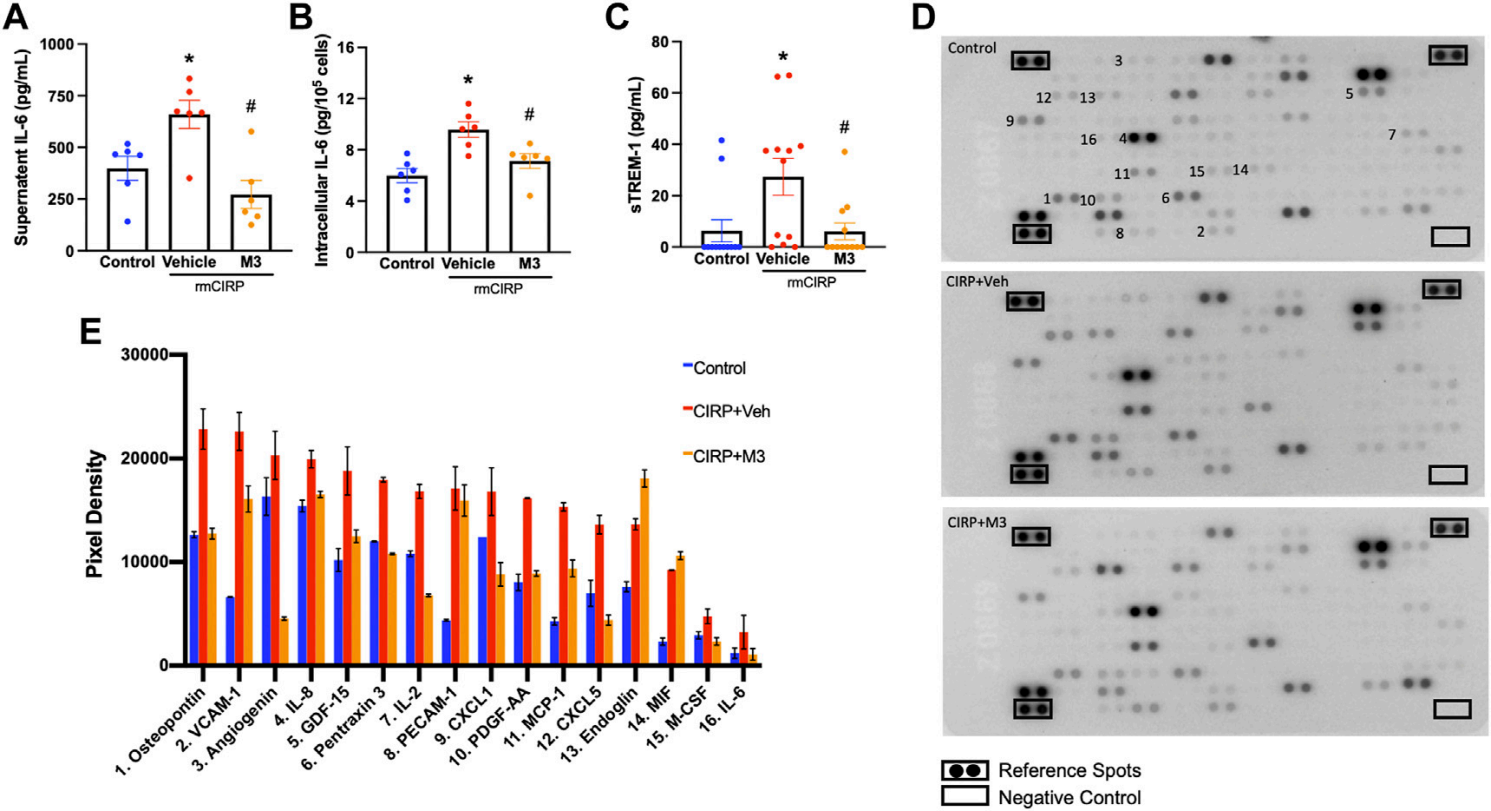
M3 attenuates rmCIRP-induced renal endothelial cell activation. HRGEC were treated with 10 μg/ml of M3 or PBS vehicle for 30 min, then stimulated with 1 μg/ml of rmCIRP. After 20 h, IL-6 protein in the **(A)** supernatant and **(B)** intracellularly was measured using ELISA. **(C)** sTREM-1 in the supernatant was measured using ELISA, *n* = 6–10 samples per group. **(D)** Relative secretion of 105 cytokines, chemokines, and growth factors in the supernatant were visualized in duplicate using a cytokine array and **(E)** pixel densities of upregulated factors were measured, *n* = 1 sample per group. All groups were compared by one-way ANOVA and Tukey’s method. *p < 0.05 vs. control, ^#^ *p* < 0.05 vs. vehicle. Data are expressed as mean ± SEM.

Activation of renal endothelial cells *via* the eCIRP/TREM-1 pathway was further studied using a cell line of human renal glomerular endothelial cells (HRGEC). Plated HRGEC were stimulated with rmCIRP, and a subset were pretreated with M3, an inhibitory peptide of TREM-1. Cells and supernatants were collected 20 h after stimulation. HRGEC stimulated with rmCIRP released 65.4% more IL-6 into the supernatant and had a 60.1% increase in intracellular IL-6 protein compared to untreated cells (Figures 4A,B). Additionally, rmCIRP induced a 4.3-fold increase in sTREM-1 release in HRGEC (Figures 4C). Incubation with M3 reduced both IL-6 protein excretion and intracellular expression in HRGEC by 58.7% and 25.6%, respectively (Figures 4A,B). Additionally, HRCEG treated with M3 had decreased activation of TREM-1, represented by a 77.7% reduction in sTREM-1 release compared to cells stimulated with CIRP alone (Figures 4C). CIRP activation of endothelial cells was further demonstrated using a cytokine array to analyze the supernatant of simulated HRGEC. Notably secretion of VCAM-1, proinflammatory cytokines including IL-2 and IL-6, immune cell chemoattractants including CXCL1, CXCL5, MCP-1, IL-8, and PDGF-AA, and proangiogenic factors including osteopontin and angiogenin were increased in cells stimulated with CIRP. Cells treated with M3 had reduced secretion of these factors (Figures 4D,E). These data suggest that eCIRP induces AKI through activation of TREM-1 expressed on renal endothelial cells.

## Discussion

**FIGURE 5:**
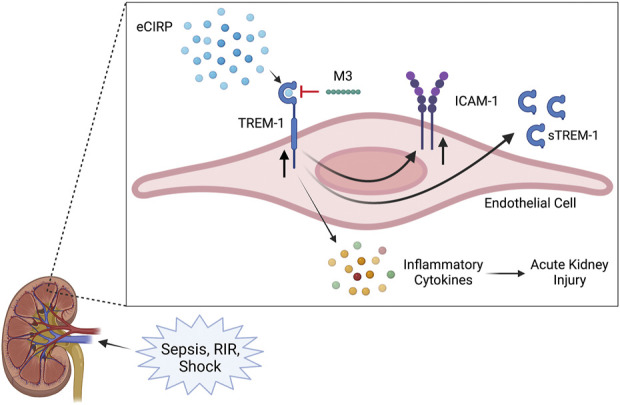
Summary of findings. Sepsis and renal ischemia/reperfusion cause an increased release of eCIRP. The circulating eCIRP interacts with TREM-1 on endothelial cells, leading to increased expression of inflammatory cytokines, upregulating TREM-1 and ICAM-1 expression, and releasing sTREM-1. This activation leads to excessive inflammation and renal tissue injury and decreasing renal function, resulting in AKI. M3, a novel inhibitory peptide of the eCIRP-TREM-1 pathway, attenuates the eCIRP-dependent activation of renal endothelial cells, leading to decreased inflammatory cytokine release and improved AKI. Created with BioRender.

Acute kidney injury encompasses a wide range of conditions leading to a sudden decrease in renal function, characterized by a decrease in glomerular filtration rate and urine output, and an increase in serum creatinine (Rewa and Bagshaw, 2014; Makris and Spanou, 2016; Levey and James, 2017; Peerapornratana et al., 2019). Although sepsis is one of the most common precipitators of AKI, its pathophysiology is multifactorial and there are currently no specific interventions for treatment (Makris and Spanou, 2016; Peerapornratana et al., 2019). During sepsis, in addition to decreased blood flow to the kidneys, there is also inflammation, microvascular dysfunction, and tubular cell injury, leading to the release of cytokines and DAMPs, further amplifying the inflammatory response (Makris and Spanou, 2016). eCIRP is a DAMP released during sepsis and is predictive of sepsis severity, associated AKI, and overall mortality (Qiang et al., 2013; Zhou et al., 2015; Zhang et al., 2018). eCIRP was recently explored as an endogenous ligand of TREM-1, capable of inducing a sepsis-like inflammatory response and propagating an inflammatory cascade in murine models of polymicrobial sepsis (Denning et al., 2020a; Denning et al., 2020b). TREM-1 activation has long been studied as a player in the dysregulated immune response in sepsis and, in its soluble form, is also predictive of sepsis severity, mortality, and development of sepsis-induced AKI (SA-AKI) (Derive and Gibot, 2011; Su et al., 2011; Brenner et al., 2017; Jedynak et al., 2018). The interplay of this ligand and receptor had never been evaluated as a potential mechanism for the development of AKI. In this study, we identified that circulating eCIRP can induce AKI *via* activation of TREM-1 by showing that mice genetically deficient in the TREM-1 receptor have attenuated renal dysfunction after intravenous administration of rmCIRP. We further demonstrate that eCIRP specifically activates renal endothelial cells *via* TREM-1, and using M3, a peptide that inhibits this interaction, decreased HRGEC activation *in vitro*. Our overall findings are summarized in Figure 5.

We have previously demonstrated that intravenous administration of eCIRP is sufficient to induce systemic inflammation and lung injury (ALI) (Yang et al., 2016). However, mice injected with rmCIRP and treated with LP17, an inhibitory peptide of TREM-1, had significantly lower levels of serum cytokines, and decreased inflammation, lung tissue injury, and endothelial activation in the lungs (Gibot et al., 2004; Denning et al., 2020a). Furthermore, TREM-1^−/−^ mice injected with rmCIRP also had decreased systemic and pulmonary inflammation (Denning et al., 2020a). We have also previously shown that inhibiting eCIRP in mice subjected to the cecal ligation and puncture (CLP) model of polymicrobial sepsis resulted in attenuated SA-AKI, as indicated by decreased serum BUN and creatinine, and NGAL and KIM-1 expression in the kidneys (Zhang et al., 2018). We therefore hypothesized that, analogous to ALI, injecting mice with rmCIRP would be sufficient to cause AKI. Indeed, we found that eCIRP induced AKI.

We also identified that TREM-1 mRNA expression was upregulated in the kidneys and sTREM-1 was increased in the serum and urine of mice with eCIRP-induced AKI, indicating that the eCIRP/TREM-1 pathway may be a novel mechanism for the development of AKI. To date, both serum and urine sTREM-1 have been implicated as diagnostic and predictive markers for SA-AKI in critically ill patients, providing further evidence of eCIRP’s role in the induction of AKI (Su et al., 2011; Dai et al., 2015). However, it remains unknown where the urinary sTREM-1 originates from (Derive and Gibot, 2011). The glomeruli in a functioning kidney freely filter proteins with a molecular weight (MW) less than 15 kDa and rapidly filter proteins up to 45 kDa (Schenk et al., 2008; Jia et al., 2009). Since sTREM-1 is a 27-kDA protein, it is possible that systemic sTREM-1 is rapidly filtered by the glomeruli and excreted by in the urine, reflecting remote production of the protein. It could also be produced locally in the kidneys by the infiltrating inflammatory cells or activated endothelial cells in response to cellular damage in AKI (Derive and Gibot, 2011; Su et al., 2015). Using a human primary cell line of glomerular endothelial cells, we have demonstrated that, when activated with CIRP, endothelial cells release sTREM-1. To our knowledge, this is the first piece of evidence demonstrating that renal endothelial cells release sTREM-1 and, therefore, could be the source of urinary sTREM-1 in AKI.

TREM-1 is expressed in many kidney cells, including renal mesangial cells (Zhao et al., 2018), tubular epithelial cells (Maiese et al., 2019; Pan et al., 2021), and kidney macrophages (Campanholle et al., 2013; Zhang et al., 2019a; Zhang et al., 2019b). TREM-1 is also expressed in endothelial cells (Gibot et al., 2019), although the role of TREM-1 in renal endothelial cells is not very well known. We chose to study renal endothelial cells in particular because during sepsis and hemorrhagic shock the blood levels of eCIRP are increased (Qiang et al., 2013; Zhou et al., 2015; Zhang et al., 2018). Once eCIRP is released into the circulation (which we have modelled by intravenous injection), endothelial cells are the first cells to have direct contact with the eCIRP in the blood. Indeed, we have previously demonstrated that intravenous administration of eCIRP induces systemic inflammation and lung injury (ALI) *via* endothelial cell activation (Yang et al., 2016).

Endothelial cells are one of the many affected cell types during SA-AKI. Immune cells, tubular epithelial cells (TECs), and endothelial cells all display PRRs, including Toll-like receptors (TLRs) and TREM-1, that are activated by DAMPs and pathogen associated molecular patterns (PAMPs), released from invading pathogens (Jolly et al., 2018; Gibot et al., 2019; Peerapornratana et al., 2019; Tammaro et al., 2019; Denning et al., 2020a). Endothelial cell activation promotes the adhesion of platelets, resulting in increased risk of thrombi formation and decreased flow, and transmigration of leukocytes, causing increased inflammation (Verma and Molitoris, 2015; Peerapornratana et al., 2019). Endothelial activation also leads to increased vascular permeability, causing interstitial edema and increasing the oxygen diffusion distance to the TECs, further aggravating the acute tubular injury (ATI) towards acute tubular necrosis (ATN) (Verma and Molitoris, 2015; Peerapornratana et al., 2019). eCIRP has been demonstrated to activate endothelial cells, increasing expression of cell-surface adhesion molecules of murine lung vascular endothelial cells (Yang et al., 2016). Additionally, septic mice treated with an eCIRP inhibitor had attenuated endothelial activation, demonstrated by decreased E-selectin and ICAM-1 expression in the lungs and kidneys (Zhang et al., 2018). Interestingly, TREM-1, which was originally identified with high expression on neutrophils, monocytes, and macrophages, has recently been identified on endothelial cells (Bouchon et al., 2000; Jolly et al., 2018). Mice deficient in endothelial TREM-1 or treated with a TREM-1 inhibitor were protected against endothelial dysfunction in sepsis (Jolly et al., 2018). Human pulmonary microvascular endothelial cells (HPMEC) stimulated with LPS *in vitro* had decreased activation and release of inflammatory cytokines after treatment with a TREM-1 inhibitor (Gibot et al., 2019). Further, maortas and mesenteric arteries isolated from TREM-1^−/−^ mice were protected from vascular dysfunction after stimulation with neutrophil extracellular traps (NETs), nuclear DNA expelled from activated neutrophils that propagate the inflammatory response in sepsis (Czaikoski et al., 2016; Boufenzer et al., 2021). Both eCIRP and TREM-1 are upregulated during sepsis, leading to endothelial cell activation and dysfunction, but their interaction has not been studied in this cell type, specifically as a contributory factor to SA-AKI.

Our work continues to explore the role of endothelial cell TREM-1 activation, specifically in the progression of AKI by showing that mice deficient in TREM-1 have attenuated endothelial activation after CIRP-induced AKI. We also show that HRGEC stimulated with CIRP have increased secretion of proinflammatory cytokines including IL-2 and IL-6, immune cell chemoattractants including CXCL1, CXCL5, MCP-1, IL-8, and proangiogenic factors including osteopontin and angiogenin. When TREM-1 was inhibited on these cells using M3, secretion of these factors was reduced. These findings provide insight to a possible novel mechanism for AKI development. Similar to pro-inflammatory cytokines such as TNF-α and IL1-β, circulating eCIRP binds to TREM-1 and stimulates renal endothelial cells, increasing expression of VCAM-1 and ICAM-1 and chemokine release. These work in tandem to recruit and home monocytes and neutrophils to the activated endothelium, and induce chemotaxis (Reitsma et al., 2007; Deshmane et al., 2009; Furuichi et al., 2009; Jourde-Chiche et al., 2019). The influx of immune cells results in further inflammation and release of inflammatory cytokines, in addition from the ones released from the endothelial cells themselves (Verma and Molitoris, 2015; Jourde-Chiche et al., 2019). In response to endothelial cell damage, proangiogenic factors like angiogenin are released and play a critical role in tissue adaptation and recovery from the AKI (Kang et al., 2002; Li and Hu, 2012; Mami et al., 2016).

Although we have focused our investigation on eCIRP/TREM-1 activation of endothelial cells and their potential contribution to AKI, eCIRP may also promote AKI by acting on other renal cells known to express TREM-1 such as renal mesangial cells (Zhao et al., 2018), tubular epithelial cells (Maiese et al., 2019; Pan et al., 2021), and kidney macrophages (Campanholle et al., 2013; Zhang et al., 2019a; Zhang et al., 2019b). However, we have not explored the possible effects of eCIRP/TREM-1 activation of these cells. Neither have we directly injected eCIRP in the renal arteries to exclude the effects of other circulating factors that eCIRP might have induced elsewhere.

In summary, we have demonstrated that systemic eCIRP is sufficient to induce AKI, and that genetic depletion of TREM-1 exhibits protective effects against the development of AKI following CIRP injection. We additionally show that renal endothelial cells are activated in CIRP-induced AKI, and TREM-1 deletion or inhibition decreases this activation. These findings provide insight into a novel pathway of endothelial cell activation involved in AKI development. Future work should focus on further characterizing the effects of CIRP/TREM-1 pathway on renal endothelial cell activation using models for sepsis and exploring M3 as a treatment for SA-AKI.

## Data Availability

The raw data supporting the conclusions of this article will be made available by the authors, without undue reservation.
